# Risk factors for small vessel disease in adults with atherosclerosis: a systematic review and meta-analysis

**DOI:** 10.1186/s12872-026-05586-2

**Published:** 2026-02-05

**Authors:** Longdi Yao, Chunlian Li, Qiang Zhu, Wenjing Xu, Xiang Li, Min Cheng, Qingwen Liu, Tiran Zhang, Yulong Wang, Tianlang Pei, Yuming Chen, Jianwen Wang

**Affiliations:** 1 Department of Thyroid and Breast Surgery, Changxing Hospital of Traditional Chinese Medicine, Huzhou, China; 2 Department of General Surgery, Northern Jiangsu People’s Hospital Affiliated to Yangzhou University, Jiangsu Province Yangzhou, China; 3 Department of Nephrology, Xinghua People’s Hospital Affiliated to Yangzhou University, Xinghua, Jiangsu Province China; 4 Department of Medical Oncology, Xinghua People’s Hospital Affiliated to Yangzhou University, Xinghua, Jiangsu Province China; 5 Department of Neurology, Xinghua People’s Hospital Affiliated to Yangzhou University, Xinghua, Jiangsu Province China; 6 Department of General Surgery, Xinghua People’s Hospital Affiliated to Yangzhou University, Xinghua, Jiangsu Province China; 7 Medical College, Nantong University, Nantong, Jiangsu Province China; 8 Department of Urology, Xinghua People’s Hospital Affiliated to Yangzhou University, Xinghua, Jiangsu Province China

**Keywords:** Small vessel disease, Atherosclerosis, Hypertension, Carotid intima-media thickness, Diabetes mellitus, Meta-analysis

## Abstract

**Introduction:**

Atherosclerosis is marked by irregular intimal plaques, known as atheroma, which intrude into the lumen of medium-sized and large arteries. Small vessel disease (SVD) is a major contributor to stroke and cognitive impairment and frequently coexists with atherosclerosis due to shared vascular risk factors and overlapping pathophysiological mechanisms. This meta-analysis aimed to systematically evaluate the association between atherosclerotic risk factors and the development of SVD in adult and elderly populations.

**Method:**

A systematic review and meta-analysis were conducted in accordance with PRISMA 2020 guidelines. PubMed, ScienceDirect, Elsevier, and Google Scholar were searched for eligible studies published between January 2000 and October 2023. Community-based randomized controlled trials and observational studies assessing atherosclerotic risk factors and SVD-related outcomes, primarily carotid intima-media thickness and carotid plaque, were included. Data were pooled using a random-effects model. Heterogeneity was assessed using the I² statistic, and publication bias was evaluated using funnel plots and sensitivity analyses.

**Results:**

Across 11 studies with sample sizes ranging from 50 to 5,585 participants, age and hypertension consistently demonstrated the strongest associations with SVD. Age showed an odds ratio of 1.07 (95% CI: 1.03–1.11; *p* = 0.0001), while hypertension yielded a pooled odds ratio of 2.24 (95% CI: 1.90–2.64; *p* < 0.0001). Diabetes was also significant (OR 1.38; *p* < 0.0001), whereas metabolic syndrome was not (*p* = 0.69).

**Conclusion:**

The study concludes that hypertension, age, diabetes mellitus, and carotid atherosclerotic markers—particularly carotid plaque—are significant predictors of SVD.

## Introduction

The accumulation of fats, cholesterol, and other substances occurs within the walls of arteries [1]. Cholesterol plaque buildup in arterial walls can lead to blood flow obstruction [2]. Rupture of these plaques may result in the sudden blockage of the artery due to clot formation. A chronic inflammatory disease in which plaque accumulates inside the arteries is called Atherosclerosis. Atherosclerosis typically remains asymptomatic until a plaque ruptures or the accumulation becomes significant enough to impede blood flow [3]. Atherosclerosis refers to the varying conditions that result in the thickening of the wall and the minimization of the elasticity of the walls of the artery [1–3]. This is very severe, and it is significant for Atherosclerosis, which can provide various underlying conditions like coronary artery disease (CAD) and cerebrovascular disease [4]. Various other factors can cause arteriolosclerosis, including Mönckeberg arteriosclerosis [5, 6]. The evaluation of cardiovascular disease risks can be observed in the Framingham Heart Study, where the main objective was to predict coronary heart disease risk. The outcome from the Third Adult Treatment Panel of the National Cholesterol Education Program have revealed the application of the risks in case of the clinical care organization, which comprised of different category of people, where low which was < 10%, the intermediate 10–20% and the high which is greater or equal to 20% have been seen in case of the 10-year risk of coronary heart disease [7, 8].

Framingham has reported different cardiovascular incidence scores. Framingham has reported different scores for cardiovascular incidence; these events include subsequent heart failure or stroke, which can be fatal, and well as non-fatal consequences. These scores, available with or without incorporating laboratory measures, serve as a comprehensive tool for risk assessment [9]. In that case, the 2008 Framingham risk score equations for primary care might be the most suitable, as presented by D’Agostino and colleagues. These scores predict total cardiovascular disease and are available in versions that include total and HDL cholesterol, as well as versions without it, using body mass index instead [10].

Atherosclerosis is marked by irregular intimal plaques, known as atheroma, which intrude into the lumen of medium-sized and large arteries [11]. These plaques comprise lipids, inflammatory cells, smooth muscle cells, and connective tissue. Different risk factors were observed, including the condition of diabetes, smoking, the pre-clinical history of family, obesity, and hypertension. Various signs and symptoms have enhanced the formation of the plaque and have reduced the flow of blood. Individual targeted specific symptoms also depend on the artery [12]. Although age-related mortality associated with atherosclerosis has seen a decline, cardiovascular disease, predominantly driven by coronary and cerebrovascular atherosclerosis, was responsible for approximately 18 million deaths globally in 2019, constituting over 30% of all deaths [13]. It has been found that patients with CAD and multiple-locus atherosclerosis were more likely to have specific genetic polymorphisms and risk factors such as smoking, diabetes, and a family history of cardiovascular diseases [14, 15]. Haverich et al. (2019) highlight the role of dysfunctional microvasculature in atherosclerosis and suggest that impaired microcirculation may be a unifying factor underlying various risk factors [16]. Kullo et al. (2000) discuss novel risk factors for atherosclerosis, including homocysteine, fibrinogen, platelet reactivity, and inflammatory markers [17].

Campeau et al. (1984) examined the progression of atherosclerosis in patients who underwent bypass surgery and found that higher LDL cholesterol and lower HDL cholesterol levels were associated with the development of new lesions [18]. Small vessel disease (SVD) is a pathologic condition characterized by damage to brain capillaries, small end arteries, venules, and arterioles. SVD and atherosclerosis most commonly co-exist, reflecting vascular risk factors and overlapping pathophysiology [11]. Endothelial dysfunction impairs nitric oxide production, increasing vascular stiffness and damaging the brain and blood-brain barrier. This promotes ischemic injury and results in white matter damage. Oxidative stress causes inflammation and microvascular remodeling, with thickened walls, resulting from active macrophage infiltration and cytokine release. The formation of Microthrombi, a change in vascular smooth muscle reactivity, and impaired autoregulation are essential in extending the initial vascular dysfunction into a more detectable form of SVD. One of the factors is age: research demonstrates significantly increased ratios of comorbid atherosclerosis in older SVD patients (prevalence rates often run into the 60–70% range in older age groups) [12].

This study aimed to conduct a meta-analysis to evaluate the significance of various risk factors in the progression of SVD in patients with atherosclerosis. It seeks to provide a comprehensive understanding of the impact of hypertension, smoking, and other key factors on the development of arterial plaques. This study has conducted a meta-analysis of global studies, identifying and comparing risk factors for SVD across populations. Data extraction across different study designs provides a clear understanding of the factors and their contributions to plaque formation, including age, sex, hypertension, and carotid plaques. This study could help to identify risk factors for the development of SVD and show the impact of the comparison.

## Methods

### Research design

This systematic review and meta-analysis were conducted from June 2025 to December 2025 in accordance with the Preferred Reporting Items for Systematic Reviews and Meta-Analyses (PRISMA) 2020 guidelines. A comprehensive literature search was performed across PubMed, ScienceDirect, Elsevier, and Google Scholar to identify relevant studies published between January 2000 and October 2023. Additional records were identified by manually screening the reference lists of included articles. The search strategy was developed using Medical Subject Headings terms and free-text keywords related to SVD and atherosclerosis. All identified records were imported into reference management software, and duplicates were removed. Two independent reviewers screened titles and abstracts for eligibility. Full-text articles were then assessed independently for inclusion. Any disagreements were resolved through discussion and consensus. The study selection process is illustrated using a PRISMA flow diagram. Data were independently extracted by two reviewers using a standardized data extraction form. Extracted information included first author, year of publication, country, study design, sample size, population characteristics, assessed risk factors, SVD definition, imaging modality, and reported effect estimates. Any discrepancies were resolved by consensus. The primary outcome was the presence or progression of SVD, assessed through neuroimaging markers such as white matter hyperintensities, lacunar infarctions, or composite SVD scores. The secondary outcomes included associations between SVD and individual atherosclerotic risk factors, including age, hypertension, diabetes mellitus, dyslipidemia, smoking status, carotid plaque, and carotid intima-media thickness. The methodological quality of included observational studies was assessed using the Newcastle–Ottawa Scale. Studies were evaluated based on selection, comparability, and outcome domains. Studies scoring ≥ 7 points were considered high quality.

### Information sources and search strategy

The authors searched ScienceDirect, PubMed, Elsevier, and Google Scholar for studies on carotid atherosclerosis risk factors and their association with minor vascular diseases. Only the studies published from 2000 to October 2023 were considered. The keywords or syntax used by the authors were “risk factors” OR “risks” AND “atherosclerosis” OR “plaque formation” AND “small vessel disease” OR “vascular disorder.” In addition to the major databases, several grey literature sources were included to reduce publication bias. Google Scholar was used to screen several studies from the available conference proceedings. Several clinical trial registries, such as ClinicalTrials.gov and the WHO International Clinical Trials Registry Platform (ICTRP), were searched for any record of unpublished studies relevant to the given topic. Several lists of references and early published studies were screened manually to ensure the study’s validity.

- *➢ P*: Patients with atherosclerosis.
- *➢ I*: Various risk factors (e.g., hypertension, smoking, metabolic syndrome).
- *➢ C*: Healthy controls or populations without atherosclerosis.
- *➢ O*: Development of SVD, assessed through outcomes like carotid intima-media thickness (CIMT) or plaque development.

### Study selection

The screening and selection of studies were performed in two stages: screening of the title and abstract, followed by review of the full text. Two independent reviewers conducted the selection process using the specified criteria. If proper agreements were not received, a third reviewer was appointed to make the final decision.

### Inclusion

- Randomized Controlled Trials and Cross-sectional studies.
- Populace residing in a particular community.
- Studies published after 2000.
- Risk and protective factors were effectively analyzed.
- A group of healthy people was used as a control, implying the absence of atherosclerosis among them.

### Exclusion

- Studies with inconsistent data.
- A study that analyses more than one risk factor is needed.
- Studies that did not analyze SVD.
- Populations outside communities.
- Non-modifiable exposures are the primary focus.
- Carotid atherosclerosis research needs a healthy control group.

### Data extraction

Data extraction was undertaken independently by two reviewers using a predesigned and piloted standardized extraction sheet to ensure methodological consistency across included studies. From each eligible article, bibliographic details, study design, country and setting, sample size, age distribution, diagnostic criteria for atherosclerosis, definition of SVD, imaging modality, and duration of follow-up were recorded. Detailed information on individual risk factors, including age, hypertension, diabetes mellitus, dyslipidaemia, smoking status, carotid intima–media thickness, and carotid plaque characteristics, was systematically captured along with reported effect estimates such as odds ratios, risk ratios, or hazard ratios and their corresponding 95% confidence intervals (CI). Any discrepancies between reviewers were resolved through discussion and consensus, with reference to the original publication to ensure the accuracy and completeness of the extracted data.

### Outcome assessment

The primary outcome was the presence or progression of cerebral SVD, as defined in the original studies. Outcome assessment was based predominantly on neuroimaging markers identified through magnetic resonance imaging or computed tomography, including white matter hyperintensities, lacunar infarctions, cerebral microbleeds, enlarged perivascular spaces, or validated composite SVD scores. In studies focusing on macrovascular correlates, surrogate markers such as carotid plaque burden and carotid intima–media thickness were accepted as indicators of vascular pathology contributing to SVD. Where multiple outcome definitions were reported, the most comprehensive and clinically relevant measure was prioritised to maintain comparability across studies.

### Risk of bias assessment

Methodological quality and risk of bias were assessed independently by two reviewers using validated tools appropriate to the study design. Observational cohort and case–control studies were evaluated using the Newcastle–Ottawa Scale, which examines the selection of participants, comparability of study groups, and adequacy of outcome assessment. Cross-sectional studies were appraised using structured risk of bias instruments addressing selection, measurement, and confounding domains, while non-randomised studies were additionally assessed with Risk of Bias In Non-randomized Studies of Interventions (ROBINS-I) where applicable. Each study was categorised as having low, moderate, or high risk of bias based on predefined criteria, and disagreements were resolved through consensus. The results of these assessments were incorporated into the interpretation of the findings and informed sensitivity analyses to assess the stability of the pooled estimates.

### Statistical analysis

Statistical analyses were performed using Review Manager. Meta-analyses’ odds ratios with 95% CI were calculated using a random-effects model, given the anticipated clinical and methodological heterogeneity among studies. Statistical heterogeneity was assessed using the I² statistic, with values > 50% indicating substantial heterogeneity. Sensitivity analyses were conducted using a leave-one-out approach, and subgroup analyses were performed by individual risk factor. Publication bias was evaluated using funnel plots.

## Results

**Fig. 1:**
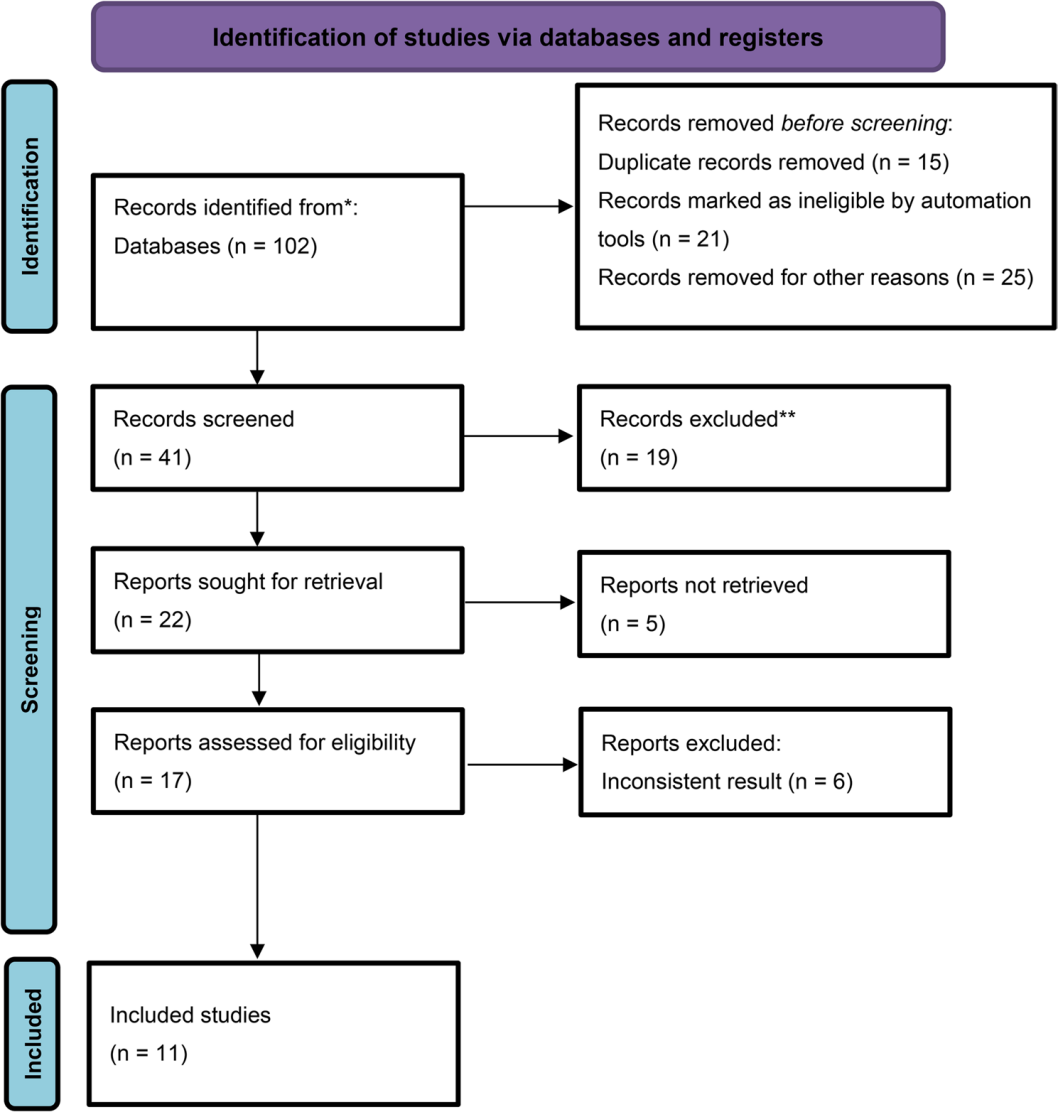
PRISMA flowchart of this meta-analysis

The search retrieved 102 studies initially, and then, due to duplicate results, ineligibility, and other reasons, 61 studies were excluded, with the remaining 41 studies included. Again, for several reasons, such as the absence of reports, 24 studies were removed, and 17 were considered. Exclusion of 6 studies was performed due to inconsistencies, including incomplete outcome reporting, unclear methodology, lack of statistical information for effect size calculation, and outcome assessment procedures that did not align with the criteria. Due to inconsistent data, six studies were excluded, and the meta-analysis considered 11 studies. Figure 1 shows the PRISMA flowchart.

### Characteristics of the included studies

The analysis includes the studies’ characteristics, presented in Table 1, and the parameters, such as study setting, design, patient age, and sample size, are indicated. The studies are different in their design, and they vary from cohort studies (e.g., O’Flynn et al. [19]) to cross-sectional studies (e.g., Sato et al. [20]). The sample sizes also varied a lot, starting from a very small one of 50 participants [19] and going up to a large one of 5,585 [21]. The study populations are predominantly middle-aged and older adults, and the participants’ ages ranged from 19 years [20] to 70 years [22], indicating a wide range of risk factors for SVD. In summary, the studies were conducted across Europe, Asia, and the Middle East, thereby making the findings applicable worldwide.

**Table 1:** Characteristics of the included studies

Ref.	Study Setting and Country	Study Design	Age of the Patients	Number of Patients
O’Flynn et al. [19]	Cork, Ireland	Observational, cohort study	60 ± 6 years	50
Al-Nidawi et al. [23]	Bahrain	Retrospective, cohort study	60.64 ± 14.95 years	477
Empana et al. [21]	France (Bordeaux, Dijon, Montpellier)	Prospective cohort study	65 to 85 years	5585
Leng et al. [24]	Hong Kong	Community-based, cross-sectional study	55.1 ± 10.4 years	653
Li et al. [25]	China (APAC Study)	Cross-sectional study	Not provided (Middle-aged adults)	2860
Han et al. [22]	China (ICASMAP & CAMERA studies)	Case-control study	57.1 ± 11.1 (symptomatic), 70.1 ± 8.4 (asymptomatic)	573
Su et al. [26]	Taiwan (Chin-Shan Community)	Community-based study	50–86 years (mean 65.7 ± 9.0)	533
Zhu et al. [27]	China (General Hospital of Northern Theatre Command)	Retrospective cohort study	62.78 ± 9.38 years	380
Sato et al. [20]	Japan (Nippon Medical School Hospital)	Cross-sectional study	19–86 years	236
Yang et al. [28]	China (Kailuan Study, Hebei Province)	Cross-sectional study	≥ 40 years	2,919
Woo et al. [29]	Korea (Community welfare centres)	Population-based screening study	50–100 years	3,030

### Comparison of risk factors in the included studies

Table 2 compares the risk factors examined across the studies. The studies focus on a wide range of risk factors associated with atherosclerosis and SVD. For example, O’Flynn et al. [19] investigate factors such as night-time systolic blood pressure, CIMT, and carotid plaques. In contrast, Empana et al. [21] assessed metabolic syndrome and blood pressure. Some studies, like Han et al. [22], compare symptomatic versus asymptomatic intracranial atherosclerotic stenosis (ICAS), while others, like Woo et al. [29], focus on a broader set of risk factors, including hypertension and smoking. The studies involve varied patient groups, ranging from those with metabolic syndrome to those with carotid plaques or hypertension. The diverse risk factors and comparative groups provide a comprehensive look at the factors influencing SVD risk.

**Table 2:** Grouping and risk factors identified for each included study

Ref.	Comparison	Number of Patients in the Study Group and the Comparator Group	Risk Factors of Atherosclerotic Patients or Patients with Arterial Changes (Plaque Formation) in Developing SVD
O’Flynn et al. [19]	Normotension, Isolated nocturnal hypertension, Isolated daytime hypertension, Day–night hypertension	50 (study group), 2047 (total cohort)	Night-time systolic BP, CIMT, Global longitudinal strain, Carotid plaques
Al-Nidawi et al. [23]	ICAS vs. Non-ICAS	123 (ICAS), 354 (non-ICAS)	Hypertension, Diabetes, Dyslipidemia, Stroke/transient ischemic attack (TIA) History, Extracranial Atherosclerotic Stenosis (ECAS)
Empana et al. [21]	MetS vs. No MetS	674 (MetS), 4911 (No MetS)	Metabolic Syndrome (MetS), Elevated blood pressure, Elevated triglycerides, Elevated waist circumference, Low HDL, High LDL
Leng et al. [24]	Metabolic Syndrome vs. No Metabolic Syndrome	188 (Metabolic Syndrome), 465 (No Metabolic Syndrome)	Abdominal obesity, Elevated triglycerides, Low HDL, Hypertension, Impaired Fasting Blood Glucose
Li et al. [25]	Study Group: Elevated Uric Acid vs. Comparator Group: Normal Uric Acid	2860 (total cohort), divided into quartiles	Serum Uric Acid levels, and vulnerable carotid plaque prevalence
Han et al. [22]	Symptomatic ICAD vs. Asymptomatic ICAD vs. Controls	104 symptomatic ICAD, 51 asymptomatic ICAD, 418 controls	Age, Hypertension, Smoking, Diabetes, HDL, LDL
Su et al. [26]	Hypertensive vs. Normotensive	263 hypertensive, 270 normotensive	Hypertension, Smoking, Age ≥ 65, Male gender
Zhu et al. [27]	IAP vs. Non-IAP (CSVD cohort), IACP vs. Non-IACP	153 CSVD, 227 ESUS (both IAP and IACP groups)	Age, Hypertension, Smoking, Diabetes, Glycemic Control, Lacunes, WMHs, CMBs, EPVSs
Sato et al. [20]	Type 2 Diabetes with Carotid Plaque vs. without Carotid Plaque	236 total patients, 154 with carotid plaque, 82 without	Duration of type 2 diabetes, Glycated albumin, Glycated haemoglobin, BMI, Systolic BP
Yang et al. [28]	Homocysteine Levels and Carotid Plaque Stability	2,919 Chinese adults, stratified by sex and age groups	Plasma homocysteine, Age, Sex, Smoking, Hypertension, Hyperlipidemia, Diabetes
Woo et al. [29]	ACS vs. CP, Age, Hypertension, Smoking, Diabetes, Hyperlipidemia	3,030 participants, sex-distributed, with risk factor analysis	Age, Hypertension, Male sex, Smoking, Hyperlipidemia, Diabetes

### Identification of risk factors studied and their obtained OR

Table 3 presents the odds ratios (OR) for different risk factors determined in the studies. Among the risk factors, hypertension, smoking, and metabolic syndrome are the most prevalent ones and are also the most significant for SVD. For example, Han et al. [22] record the OR of 1.84 for diabetes, which is statistically significant. In contrast, Woo et al. [29] give a very high OR for smoking of 6.97, marking the strong association of smoking with SVD. Furthermore, Su et al. [26] report an OR of 5.0 for hypertension, reflecting its central role in the emergence of SVD. On the contrary, certain risk factors, such as Carotid Plaque and Metabolic Syndrome, as pointed out by Leng et al. [24], are associated with lower ORs (1.50 and 1.17, respectively). The differences in ORs among the studies emphasize the varying impacts of risk factors on SVD, with lifestyle factors like smoking and hypertension, particularly highlighting their strong relationships with the disease.

**Table 3:** Identification of risk factors studied and their obtained OR

Ref.	Risk Factors Identified	OR for each Risk factor
O’Flynn et al. [19]	Night-time systolic BP, CIMT, Global longitudinal strain, Carotid plaques	OR for CIMT: 1.15 (1.03–1.28), OR for Carotid Plaques: 1.47 (1.16–1.88)
Al-Nidawi et al. [23]	Hypertension, Diabetes, Dyslipidemia, Stroke/TIA History, ECAS	OR for Stroke/TIA: 1.91, OR for Hypertension: 1.49, OR for Dyslipidemia: 1.79
Empana et al. [21]	MetS, Elevated blood pressure, Elevated triglycerides, Elevated waist circumference, Low HDL, High LDL	OR for Elevated BP: 1.28, OR for Low HDL: 1.62, OR for High LDL: 1.32
Leng et al. [24]	Metabolic Syndrome, Abdominal obesity, Elevated triglycerides, Low HDL, Hypertension, Impaired fasting glucose	OR for Metabolic Syndrome: 1.17 (1.11–2.64) for CIMT, OR for Carotid Plaque: 1.50 (0.92–2.46)
Li et al. [25]	Serum Uric Acid levels, and vulnerable carotid plaque prevalence	OR for Uric Acid: 1.27 (1.02–1.59) for plaque development
Han et al. [22]	Age, Hypertension, Smoking, Diabetes, HDL, LDL	OR for Hypertension: 1.72 (1.21–2.45), OR for Diabetes: 1.84 (1.38–2.48)
Su et al. [26]	Hypertension, Smoking, Male gender, Age ≥ 65 years	OR for Hypertension: 5.0 (3.0 to 8.4), OR for Smoking: 4.8 (1.4 to 16.5)
Zhu et al. [27]	Intracranial atherosclerotic plaque (IAP), white matter hyperintensity (WMH), lacunes, enlarged perivascular spaces (EPVS)	OR for IAP with WMHs: 2.00 (1.03–3.90), OR for IACP with Lacunes: 4.26 (1.4–12.97)
Sato et al. [20]	Glycated Albumin (GA), Glycated Haemoglobin (HbA1c), Age	OR for GA: 1.05 (1.01–1.09), OR for HbA1c: 1.17 (1.01–1.37)
Yang et al. [28]	Plasma homocysteine, Age, Sex, Smoking, Hypertension, Hyperlipidemia, Diabetes	OR for Homocysteine: 1.28 (1.09–1.51) for advanced plaque, OR for Male: 1.41 (1.17–1.70)
Woo et al. [29]	Age, Hypertension, Male sex, Smoking, Hyperlipidemia, Diabetes	OR for Age: 1.07 (1.03–1.12), OR for Hypertension: 3.16 (1.34–7.46), OR for Smoking: 6.97 (1.78–27.31)

**Fig. 2:**
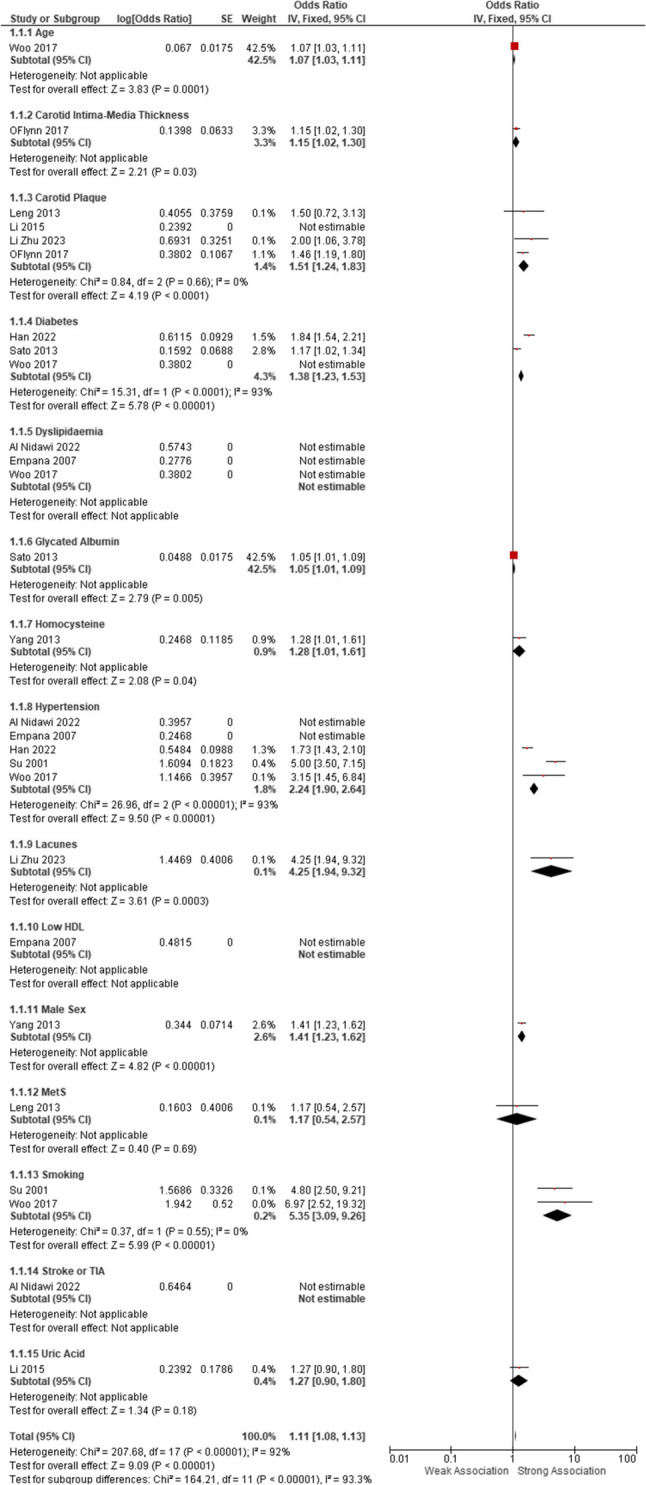
Forest Plot showing the meta-analysis of the studies with respect to the risk factor identified in developing SVD

Figure 2 presents a forest plot summarising odds ratios (ORs) for various risk factors associated with the development of SVD, drawn from multiple studies. Each risk factor is represented with its corresponding odds ratio and 95% CI, providing insights into the relative contribution of each factor to the risk of SVD. Regarding age, the study conducted by Woo et al. [29] has demonstrated an OR of 1.07 with a 95% CI of [1.03, 1.11] and a *p*-value of 0.0001, indicating that age is a crucial risk factor for SVD. The age of the subjects has been assigned a high weight (42.5%) in the study, indicating that age is a major contributor to the increased risk of SVD. Likewise, CIMT, as per the study of O’Flynn et al. [19], gives an OR of 1.15 (95% CI [1.02, 1.30], *p* = 0.03), thereby providing some evidence that an increase in CIMT comes along with an increased risk of SVD, albeit to a lesser extent compared to age. The Carotid Plaque risk factor, as shown in studies by Leng et al. [24], Li et al. [25], and Zhu et al. (2023), has an OR of 1.51 (95% CI [1.24, 1.83]). Although no *p***-**value is provided, the reliability suggested by the agreement of findings across various authors is a major contribution to SVD development, given the low heterogeneity among the studies, as evidenced by minimal variation. Diabetes mellitus is also considered a major risk factor for SVD, with an OR of 1.38 (95% CI [1.23, 1.53], *p* < 0.0001) based on the combined results from Sato et al. [20] and Li Zhou et al. (2023). This factor shows high statistical significance and a large effect size, suggesting that diabetes is a key risk factor for SVD.

Dyslipidaemia (Al-Nidawi et al. [23]; Empana et al. [21]; Woo et al. [29]), by contrast, lacks any estimable ORs across studies, and the data are difficult to interpret. The heterogeneity of risk factors cannot be applied. For Glycated Albumin, the combined OR of 1.05 (95% CI [1.01, 1.09], *p* = 0.005) from Sato et al. [20] studies further support its possible involvement in SVD, although the effect size is slight. Other risk factors, such as Homocysteine, Hypertension, and Lacunes, are showing different results throughout the studies, with hypertension [22, 29] exhibiting a notable OR of 2.24 (95% CI [1.90, 2.64], *p* < 0.0001), being a strong indicator of the association between the risk of developing SVD and its presence. Male Sex and MetS (Metabolic Syndrome) are the last to show an association with moderate effect, with ORs of 1.41 (95% CI [1.23, 1.62], *p* < 0.0001) and 1.17 (95% CI [0.54, 2.57], *p* = 0.69), respectively. The latter implication is that metabolic syndrome might not be a significant risk factor for SVD since the CI includes the value 1.

The study suggests that risk factors like age, carotid intima-media thickness, diabetes, and hypertension significantly increase the odds of developing SVD. Other factors, such as glycaemic control and carotid plaque, also show moderate associations, whereas some factors, such as dyslipidaemia and smoking, remain less conclusive in their effects. The overall data strongly point to age and hypertension as critical contributors to SVD risk.

**Fig. 3:**
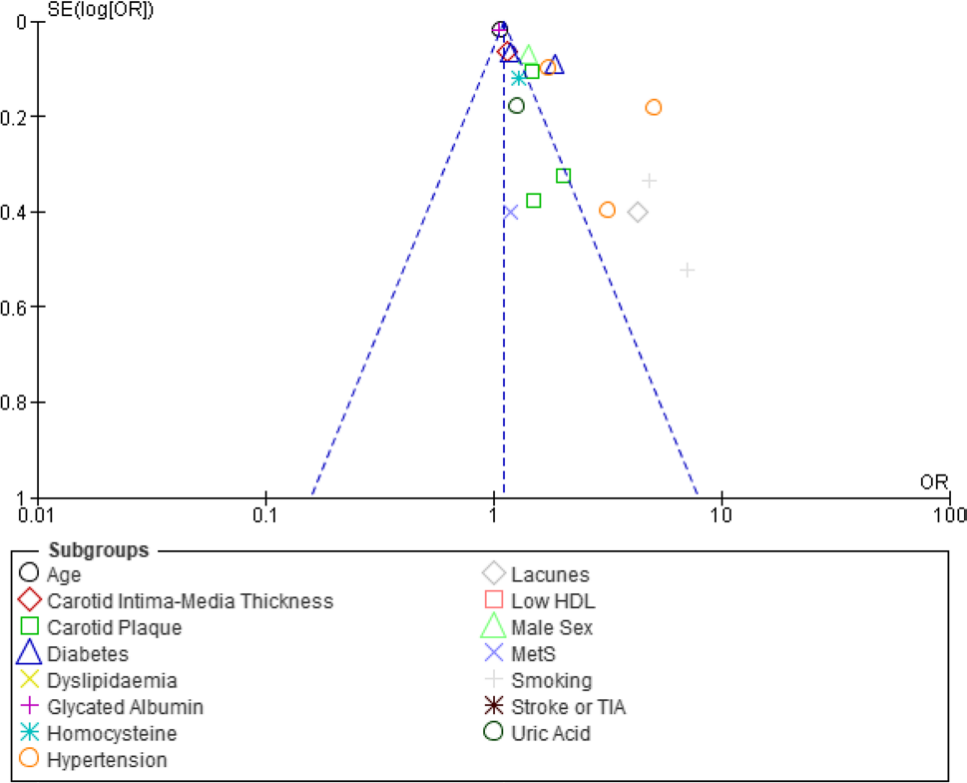
Funnel Plot showing the heterogeneity of the included studies

From Fig. 3, it was found that *Age, Carotid Intima-Media Thickness*, and *Diabetes* are the strongest risk factors, with ORs significantly greater than 1. At the same time, *Stroke or TIA* and *Uric Acid* show weaker associations. The plot reflects the precision of each estimate, with larger circles indicating more uncertainty in the OR values.

The bias assessments across the different tools indicate generally low risk of bias in most studies, with a few exceptions. Studies in Table 4A (Cross-sectional Risk of Bias) exhibit low selection and confounding biases, but performance and detection biases are often unclear. Table 5 (Newcastle-Ottawa Scale) shows low bias ratings for all studies, with robust selection and outcome assessments. Table 6 (ROBINS-I) highlights some moderate biases in missing data and reported results, whereas Table 7 (Risk of Bias Across Components and Studies (RoBACS) and Population-Based Screening) reveals moderate biases in detection and confounding for some studies. However, selection bias remains low across the board.

**Table 4:** **A**: bias assessment using the Cross-sectional risk of bias tool

Cross-sectional Risk of Bias tool				
Study	Selection Bias	Performance Bias	Detection Bias	Confounding Bias
Yang et al. [28]	Low	Unclear	Unclear	Low
Leng et al. [24]	Low	Unclear	Low	Low
Li et al. [25]	Low	Unclear	Low	Low
Sato et al. [20]	Low	Unclear	Low	Low

**Table 5:** Bias assessment using Newcastle-Ottawa scale

Newcastle-Ottawa Scale					
Study Name	Selection	Comparability	Outcome	Total Score	Bias Rating
Su et al. [26]	★★★	★★	★★★	★★★★	Low
Empana et al. [21]	★★★	★★★	★★★	★★★★	Low
O’Flynn et al. [19]	★★★	★★	★★★	★★★★	Low

**Table 6:** Bias assessment using ROBINS-I bias assessment

ROBINS-I Bias Assessment								
Study	Confounding	Selection of Participants	Classification of Exposure	Deviations from Exposure	Missing Data	Measurement of Outcomes	Selection of Reported Results	Overall Bias
Al-Nidawi et al. [23]	Low	Low	Low	Low	Moderate	Low	Low	Low
Zhu et al. [27]	Moderate	Low	Low	Low	Low	Low	Moderate	Moderate

**Table 7:** Bias assessment using risk of bias in Case-Control studies and risk of bias in Population-Based screening studies

Risk of Bias in Case-Control Studies	
Bias Domain	Risk of Bias Assessment
Selection Bias	Low
Performance Bias	Low
Detection Bias	Moderate
Attrition Bias	Low
Reporting Bias	Moderate
Confounding	Moderate

Risk of Bias in Population-Based Screening Studies	
Bias Domain	Risk of Bias Assessment
Selection Bias	Low
Information Bias	Moderate
Performance Bias	Low
Detection Bias	Low
Attrition Bias	Low
Confounding	Moderate

## Discussion

O’Flynn et al. [19] have revealed that nighttime systolic blood pressure was associated with a high incidence of carotid media thickness and carotid plaques, suggesting that nocturnal hypertension may contribute to early vascular alterations. Night-time blood pressure is a more efficient predictor of damage in the subclinical vascular region than daytime blood pressure. In our study, the results align with our meta-analysis, which identifies hypertension and CIMT as crucial risk factors for small-vessel disorder, underscoring the importance of blood pressure regulation, including nocturnal measurements, for reducing the risk of SVD [19]. Al-Nidawi et al. [23] investigated various demographic and risk factors for ICAS. The study has concluded that hypertension, diabetes, and dyslipidemia were the main risk factors, with hypertension having a connection with the ICAS. Age is a significant contributor, along with other factors like prior stroke/TIA and ECAS, which can enhance the risk profiles. The findings have revealed the primary roles of vascular and metabolic risk factors in the establishment of intracranial arterial conditions.

Our meta-analysis findings are consistent with those of the study, which found that age, hypertension, diabetes, and CIMT are risk factors for SVD. Both of the studies have stimulated the impact of hypertension and diabetes on the requirement for the detection and the management of the risk factors for the reduction of the impact of SVD [23]. Empana et al. [21] have shown an association between MetS and carotid artery structure. The study has revealed that MetS is associated with increased CIMT and an increased likelihood of arterial plaque, highlighting the effects of metabolic and vascular risk factors. Different components of MetS, such as high blood pressure, triglycerides, and waist circumference, were correlated with adverse changes in the carotid region, assessing the impact of metabolic changes. While our meta-analysis also aligned, age, hypertension, diabetes, and CIMT were the main factors for SVD. Both studies have revealed the impact of metabolism and vascular risk on arterial structure. High heterogeneity (I² = 90%) and variation in the assessment of plaque have been observed in our meta-analysis, which reveals different procedures and risk definitions that can affect the robustness [21]. Leng et al. [24] conducted a study to investigate the association between MetS and carotid atherosclerosis.

The study has revealed that patients with MetS have increased CIMT and a high likelihood of carotid plaques, indicating a close association between MetS and subclinical atherosclerosis. Different components were present in the MetS, which include high blood pressure, central obesity, and dyslipidemia. This aligns with our meta-analysis, in which age, hypertension, diabetes, and CIMT were the main factors associated with SVD. Both studies have emphasized the importance of metabolic function and vascular factors in arterial alterations. High heterogeneity (I² = 90%) was observed in our study, reflecting differences among the study population, design, and protocol [24]. Li et al. [25] examined the relationship between atherosclerotic carotid plaques and serum uric acid (SUA) levels. The study has revealed that SUA level is important for the association with an increased risk of carotid plaques, and this association is independent of conventional cardiovascular risk factors. This reveals that elevated uric acid levels increase plaque instability and perpetuate subclinical atherosclerosis. The alignment with our meta-analysis has revealed that metabolic and biochemical factors, including diabetes, changes in vascular structure, and hypertension, can stimulate SVD and plaque formation. Both studies have established the importance of the multifactorial condition of atherosclerosis, and our study reported I² = 90%, with high variation in the prevalence of plaque, the study population, the study design, and the measurement protocol [25]. Han et al. [22] studied the use of MR vessel wall imaging to identify risk factors in both asymptomatic and symptomatic ICAS. Hypertension, diabetes, and high LDL-cholesterol are associated in both cases. However, the large plaque was observed in symptomatic older individuals, and metabolic factors also influenced disease severity. In our meta-analysis, age, hypertension, and diabetes are the crucial risk factors. Our study has also shown high heterogeneity and variation in the likelihood of plaque formation. Apart from the variation, both studies have identified risk factors for atherosclerosis [22].

Su et al. [26] have revealed that hypertension is the most significant contributor to carotid atherosclerosis. People with elevated blood pressure are at high risk of CIMT and an increased risk of carotid plaque formation. Compared to our meta-analysis, hypertension and age were the main contributors to the alteration in atherosclerosis. Hypertension is the primary contributor to plaque formation [26]. Li et al. [25] reported that serum uric acid (SUA) was associated with an increased risk of carotid plaques, independent of cardiovascular factors. The high level of SUA makes the plaque unstable and contributes to the biochemical marker. Compared with our meta-analysis, metabolic and vascular factors were the main drivers of plaque development, and high heterogeneity was observed in our study [27]. Sato et al. [20] have revealed that the enhanced glycated albumin (GA) levels have been associated with type 2 diabetes. GA is related to plaque formation, whereas HbA1c reflects glycemic fluctuations and is a strong contributor to the risk of atherosclerosis. GA is a strong marker for evaluating the vascular investigation. Our study findings support the conclusion that metabolic dysregulation is a significant contributor to plaque formation. Both studies have stimulated the requirement for improved and more consistent risk factor evaluation for a better understanding of the progression of atherosclerosis [20].

Yang et al. (2014) found that homocysteine levels were strongly associated with carotid plaque instability, a key marker of plaque instability [28]. The study has revealed that homocysteine is a risk factor for unstable plaque. Our meta-analysis study is more consistent and specific, whereas heterogeneity and variation were prevalent in the plaque [28]. Woo et al. [29] have shown that 63.23% have been seen for the carotid plaque, and the age, sex, and hypertension, diabetes, and smoking were the main factors for the carotid disease. Studies have revealed a strong and consistent association in the population-based study. Our study findings also align with those of a prior study, which showed strong, consistent associations across a large population-based sample, with high variability (I² = 90–93.8%) observed [29]. The study has revealed that smoking, hypertension, diabetes, and hyperlipidemia were the main factors for the peripheral atherosclerosis. Old age and the male sex have contributed to the reduction in the risk pattern. Our study findings have aligned, where it shows that the high heterogeneity and variability among the studies included in the meta-analysis, which also show he inconsistent and more careful observation [30]. Goya et al. (2003) found that age, longer diabetes duration, higher HbA1c, hypertension, and higher LDL-cholesterol were the strongest predictors of asymptomatic atherosclerosis. The results have shown metabolic control and reduced cardiovascular disease risk, highlighting the importance of carotid atherosclerosis. Our study also has shown more consistent and specific population-based studies, with more heterogeneity and variability, which decreases the uniformity and the generalized plaque formation of the studies [31].

Grundy et al. [32] have shown that LDL-C has reduced the use of statins as first-line therapy, as well as of other agents such as ezetimibe and PCSK9 inhibitors, in high-risk groups. This study also provides a more personalized evaluation of lifestyle changes to prevent. These recommendations have highlighted greater variation in the population-level prevalence of plaque and its associated risk factors [32]. Colantonio et al. (2019) reported that adults at high risk have high rates of ischemic events. The events were associated with the LDL-C and with various comorbidities. The findings have shown the prevalence of plaques in the study process, and the plaques were due to the heterogeneous protocol for the study process [33]. Vallejo-Vaz et al. (2019) have revealed that the use of alirocumab to lower LDL-C levels reduces cardiovascular conditions in high-risk patients. The study also confirmed the association between the reduced LDL-C and its outcomes. Our study has highlighted the significance of the study design and the study population [34]. Piepoli et al. (2016) have stimulated interventions targeting lifestyle and other risk factors, and well as high and low LDL, for the prevention of cardiovascular conditions. In our study, heterogeneity has enhanced the standard process for the guidelines [35]. Mach et al. have reported that low lipid levels recommend the use of statins or add-on therapy to meet targets. This study has revealed LDL-C as the main factor for atherosclerosis. Consistent and evidence-based studies have revealed the heterogeneity in plaque formation and the associated risk factors for the research [36]. Virani et al. [37] There were also differences in plaque prevalence and in the guidelines’ recommendations, which have posed a challenge for translating the policies to a more heterogeneous population.

### Limitations of the study

This meta-analysis is limited by substantial heterogeneity among included studies, arising from differences in study design and population characteristics. This, in one way, contributed to the study’s increased generalizability. Thus, variability in imaging modalities and outcome assessment methods may affect the consistency and generalizability of the pooled estimates. In some ways, the predominance of observational and cross-sectional studies limits causal inference.

## Conclusion

The meta-analysis has concluded that the key risk factors for developing SVD include age, hypertension, carotid intima-media thickness, and diabetes. Certain factors have already been recognized as the main drivers of SVD prediction, and among these, hypertension and carotid plaque remain the strongest. The overall data emphasize these risk factors as extremely important for the development of SVD. So, to conclude, the risk factor studies for SVD have pointed out several main factors, such as age, hypertension, carotid IMT, and diabetes, which play a significant role in the risk of SVD development. The research is quite different in design and patient characteristics, providing a broad global view of the involved risk factors. Even though there were different odds ratios (ORs) reported in other studies, hypertension and carotid plaque were still very strongly associated with SVD. Moreover, the authors of the bias assessments rated the risk in most studies as low to moderate, which further supports the reliability of the findings.

## Data Availability

The data could be obtained by contacting the corresponding author.
